# Restoration of the current transformer secondary current under core saturation conditions based on ANN

**DOI:** 10.1016/j.heliyon.2024.e37960

**Published:** 2024-09-14

**Authors:** Ismoil Odinaev, Abdel-Haleem Abdel-Aty, Andrey Pazderin, Murodbek Safaraliev, Pavel Matrenin, Mihail Senyuk, Amir Abdel Menaem, Mohammad Kanan

**Affiliations:** a Department of Automated Electrical Systems, Ural Federal University, 620002, Yekaterinburg, Russia; b Department of Physics, College of Sciences, University of Bisha, Bisha 61922, Saudi Arabia; c Scientific Laboratory of Digital Twins in the Power Industry, Ural Federal University, 620002, Yekaterinburg, Russia; d Electrical Engineering Department, Mansoura University, 35516, Mansoura, Egypt; e Department of Industrial Engineering, College of Engineering, University of Business and Technology, Jeddah, 21448, Saudi Arabia; f Department of Mechanical Engineering, College of Engineering, Zarqa University, Zarqa, Jordan

**Keywords:** Current transformer, Core saturation, Protection system, Artificial neural network

## Abstract

Relay protection and emergency automation is an integral part of electric power systems. The algorithms of the relay protection and emergency automation are based on the use of analysis of the parameters of the electrical mode - current and voltage - obtained from current and voltage instrument transformers. As a rule, these transformers operate on the basis of the laws of electromagnetism. Operational experience shows that the magnetic core of current transformers under fault conditions can be saturated. As a result, the shape of the measured current is distorted which can lead to false operation of the relay protection and emergency automation system. In this paper a comparative analysis of the existing methods is presented. The advantages and disadvantages of them are described. The problem of current transformer core saturation is reduced to a regression problem. To solve the problem an artificial neural networks-based method is proposed. Computational experiments for both proposed and existing methods are performed. The results of experiments show that the efficiency of the proposed method is 1.25 times higher relative to the most effective existing method. The method of re-scaling data at real-time work applied to the restoration of the distorted shape of current is proposed.

## 1 Introduction

One of the main tasks of the exploitation of electric power systems is to identify and eliminate emergency modes that cause damage to primary equipment. To solve this problem, relay protection and emergency automation systems (RP&EA) are used [[1], [2], [3], [4]], receiving information mainly from electromagnetic current (CT) and voltage transformers. Operational experience shows that in several cases the CT core saturation can occur. As a result, the error of the measured quantity begins to grow sharply [5]. This leads to a maloperation of the algorithms of the RP&EA systems.

As a rule, under CT core saturation conditions the following may occur: false operation of the differential protections, a slowdown in the operation of backup protections and incorrect operation of the fault location algorithms [6]. In order to avoid maloperation of differential protections during CT core saturation the restraint characteristics are applied [5]. The effectiveness of their work depends on the depth of the saturation. For example, with deep saturation the effectiveness of mentioned way decreases sharply. This declines the reliability of the protection systems.

Presently to exclude the negative effect of CT saturation on the operation of the RP&EA, detection of the saturation moment and replacing the distorted section of the measured current with the theoretical one are performed [7].

Methods for the detection of the moment of CT core saturation and obtaining a theoretical current curve, depending on their implementation, can be divided into two approaches: constructive modification of CT [8,9] and mathematical signal processing [[10], [11], [12], [13], [14], [15], [16], [17], [18], [19], [20], [21], [22], [23], [24], [25], [26], [27], [28], [29], [30], [31], [32], [33], [34], [35], [36], [37], [38], [39], [40], [41], [42], [43]].

The essence of the methods proposed within the first approach is the optimization of the absolute magnetic permeability *μ*_*а*_ of the CT core. For example, the *μ*_*а*_ optimization by reducing the number of turns of the secondary winding. As a result, the transformation ratio decreases which leads to an increase in the measured quantity on the secondary side of the CT:

(1) $${\Delta I}_{N}=\frac{{\Delta N}_{2}}{N_{2r}}∙100\%\text{,}$$

where *ΔI*_*N*_ – is the deviation of the secondary current from the nominal value; *ΔN*_*2*_ – is the number of turns by which the secondary winding was reduced; *N*_*2r*_ – is the nominal number of turns of the secondary winding.

**Fig. 1:**
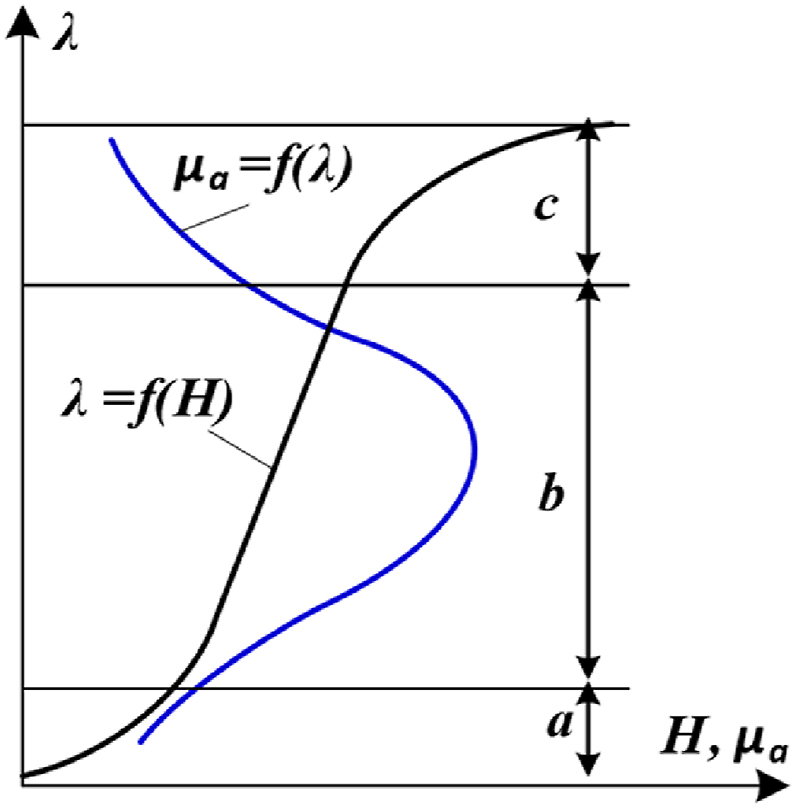
The dependence of the *μ*_*a*_ = f(λ) and the λ = f(H).

Fig. 1 shows the changing trajectory of the absolute magnetic permeability (*μ*_*a*_) in the CT core depending on the flux density (*μ*_*a*_ = f(λ), blue line) and the change of flux density on the magnetization curve of the CT (λ = f(H), black line).

It can be seen from Fig. 1 that *μ*_*a*_ reaches its maximum value when the *λ* is on a linear section of the magnetization curve (zone b). Otherwise, the value of *μ*_*a*_ drops sharply (zone a and c).

The methods of the first approach proposed in Refs. [8,9] are able to compensate for CT errors when the primary current changes within 10–120 % of the nominal value. When the CT is saturated, the use of these methods is not possible.

The methods of the second approach proposed in Refs. [[10], [11], [12], [13], [14], [15], [16], [17], [18], [19], [20], [21], [22], [23], [24], [25], [26], [27], [28], [29], [30], [31], [32], [33], [34], [35], [36], [37], [38], [39], [40], [41], [42], [43]], in turn, can be conditionally divided into two parts: techniques for compensating the CT error by replacing the distorted section of the measured current with a theoretical curve [[10], [11], [12], [13], [14], [15], [16], [17], [18], [19], [20], [21], [22], [23], [24], [25], [26], [27], [28], [29], [30], [31], [32], [33]] and techniques for detecting CT saturation [[34], [35], [36], [37], [38], [39], [40], [41], [42], [43]].

The detailed analysis of the CT error compensation methods proposed in Refs. [[10], [11], [12], [13], [14], [15], [16], [17], [18], [19], [20], [21], [22], [23], [24], [25], [26], [27], [28], [29], [30], [31], [32], [33]] will be described in next section. The methods for the detection of the CT saturation suggested in Refs. [[34], [35], [36], [37], [38], [39], [40], [41], [42], [43]] can be divided into groups:

1. Detection based on the mathematical analysis methods [[34], [35], [36], [37], [38]];
2. Detection using mathematical statistics [[39], [40], [41]];
3. Detection on the basis of the use of artificial neural networks (ANN) methods [42,43].

The CT saturation problem within [[34], [35], [36], [37], [38]] is mainly reduced to numerical differentiation. The base of the methods is that during saturation in the derivative of the measured current the outbursts occur. Based on these outbursts the detection of saturation is performed. The second order derivative is used as it suppresses the fault DC component.

The advantages of the above-mentioned methods are their speed-of-response. However, they are sensitive to the noise and harmonical components in the fault current and to the form of distorted current.

The base of the methods proposed in Refs. [[39], [40], [41]] is statistical data analysis. The authors in Ref. [39] propose to detect CT saturation using correlation analysis between measured and estimated currents. The method proposed in Ref. [40] is based on the use of the density analysis of the measured current values. When dispersion of measured current values is decreased (under saturation the values of the measured current concentrate around x-axis) the CT saturation is established. The essence of the method proposed in Ref. [41] is the dispersion analysis of the flux density. According to Ref. [41] under CT core saturation conditions, the dispersion of the flux density is rapidly decreased which is used as an indicator.

The advantages of the statistical data analysis-based methods suggested in Refs. [[39], [40], [41]] are their robustness to the noise and harmonical component in measured current. Their main disadvantage is time delay.

Within methods proposed in Refs. [42,43] the saturation problem is reduced to the binary classification which is solved by using ANN. The advantage of methods proposed in Refs. [42,43] is the robustness to the noisy and harmonic components of the measured current. Their main disadvantage is computational load and probabilistic characteristics.

The paper proposes a numerical analysis of existing ANN-based methods for current restoation and suggests a new way to scale data with a fully connected architecture of forwarded propagation ANN which allows to restore distorted current values at the rate of information receipt.

In section 2 the paper shows the analysis of both the existing deterministic- and ANN-based measured current recovery methods. The architecture of the proposed ANN with the most appropriate internal parameters and the proposed method for data preprocessing in relation to the task of restoring current are decribed in section 3 as well as the factors causing CT saturation and method for generating data considering these factors are presented in this section. Finally, the computational results are described in section 4.

## 2 The present state of the CT core saturation problem

### 2.1 Review of deterministic methods

To compensate CT error methods based on mathematical signal processing have been widely developed theoretically:

1. Restoration of the distorted current section based on the use of CT parameters [[10], [11], [12], [13]];
2. Prediction of a distorted current section based on unsaturated sections [[14], [15], [16], [17]];
3. Restoration of the distorted current section based on a combination of methods of the two previous groups [[18], [19], [20], [21], [22]];
4. Restoration of the distorted current section based on the use of ANN methods [[23], [24], [25], [26], [27], [28], [29], [30], [31], [32], [33]].

The procedure of the method proposed in Refs. [[10], [11], [12], [13]] involves the use of CT parameters, saturated and unsaturated sections of measured current. This method is denoted A1. Based on the specified parameters the flux density can be calculated as following:

(2) $$\lambda \left(t\right)=\frac{R_{2}}{w_{2}s}\int_{t_{0}}^{t}i_{2}\left(\zeta \right)∙d\zeta +\frac{L_{2}}{w_{2}s}∙\left[i_{2}\left(t\right)-i_{2}\left(t_{0}\right)\right]+\lambda \left(t_{0}\right)\text{,}$$

where *s* – is the cross section of the magnetic circuit CТ, *m*^*2*^; *R*_*2*_ and *L*_*2*_ – are, respectively, the active resistance and inductance of the secondary winding, *Ω* and *H*, respectively; *i*_*2*_ – is the measured current, *А*; *w*_*2*_ is the number of turns of the secondary winding.

Further, knowing the flux density, using the magnetization curve *λ* *=* *f(H)* to determine the magnetic field strength *H* and calculate the magnetizing current is possible:

(3) $$i_{\mu }\left(t\right)=\frac{H\left(t\right)∙l}{w_{2}}\text{,}$$

where *l* – is the average length of the magnetic path of the CT core.

Then, the restored current is:

(4) $${i_{1}^{\prime }\left(t\right)=i}_{\mu }\left(t\right)+i_{2}\left(t\right)\text{.}$$

The advantage of A1 is its robustness to harmonics and random components in the measured current, to the fluctuation of the network frequency and decay rate, to the initial value of the AC component and the parameters of the DC component of the fault. The main disadvantage of A1 is it depends on the CT parameters. When implementing the method, it becomes necessary to adapt it to a specific CT. Moreover, in the presence of remanent flux density, the use of this method is impossible.

The procedure of the method proposed in Refs. [[14], [15], [16], [17]] is to approximate the saturated section of the measured current using unsaturated one. This method is denoted A2. As an approximating function an analytical expression describing the behavior of the fault is usually used:

(5) $$i_{1}^{\prime }\left(t\right)=I_{m1}\;\sin\left(\omega t+\varphi \right)+I_{m2}\;e^{-\gamma t}\text{,}$$

where *ω* is the cyclic frequency, in this method it is considered known and is assumed to be equal to the nominal frequency of the network.

In expression (5) the unknown parameters are the amplitude *I*_*m1*_ and the initial phase *φ* of the AC component of the fault as well as the initial value *I*_*m2*_ and the decay rate *γ* of the DC component of the fault. To quickly find these parameters using trigonometric transformations and Taylor series expansion for the first order, expression (5) is reduced to the following form:

(6) $$i_{1}^{\prime }\left(t\right)={С}_{1}∙\sin\left(wt\right)+{С}_{2}∙\cos\left(wt\right)-{С}_{3}∙t+{С}_{4}\text{.}$$

Next, based on the well-known least squares method, parameters are searched in the *C*_*1*_–*C*_*4*_ space. After that, one can restore the distorted sections of the current:

(7) $$i_{1}^{\prime }\left(t\right)=A_{c}∙\sin\left(wt+\varphi_{c}\right)+{С}_{4}∙e^{\left(C_{3}/C_{4}\right)t}\text{,}$$

where $A_{c}=\sqrt{C_{1}/C_{2}}$, $\varphi_{c}=\arcsin\left(C_{2}/C_{1}\right)$, $C_{3}/C_{4}=\gamma$.

It is worth noting that the unknown parameters *C*_*1*_–*C*_*4*_ in (6) are expressed in linear form. The positive aspects of the method A2 include the absence of binding to the CT electric and magnetic parameters. This allows one to avoid additional settings when applying it to any type of CT. The main disadvantage of A2 is the difficulty of predicting changes in the transition process based on data obtained on the unsaturated section. Its other disadvantage is its sensitivity to the presence of noise and harmonics in the measured current.

**Fig. 2:**
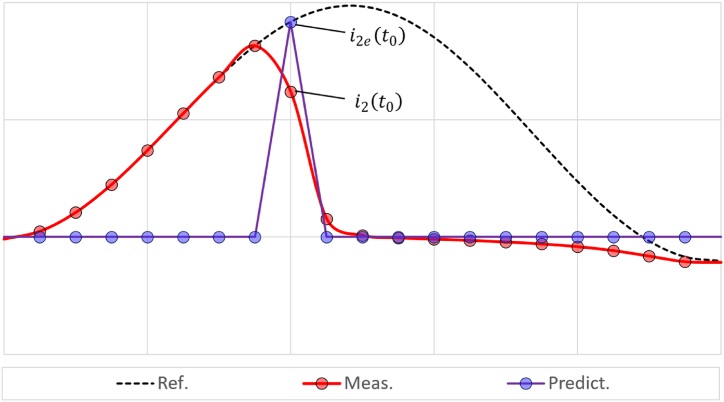
Search for initial induction using the method A3.

The essence of the method proposed in Refs. [[18], [19], [20], [21], [22]] is a combination of methods A1 and A2. Next, this method is denoted as A3. The combination of A1 and A2 allows to increase the robustness of A3 to remanent flux density in the CT core. When CT saturation occurs, one of the methods of A2 is used to predict the current value at the beginning of its distorted section. This is shown in Fig. 2. The lines with red and blue circles, respectively, correspond to the distorted and predicted current values. The dotted line shows the reference current.

After the prediction, the magnetizing current and remanent flux density are calculated based on the difference between the predicted and measured current values.

(8) $$\lambda \left(t_{0}\right)=f\left(i_{\mu }\left(i_{2}\left(t_{0}\right),i_{2e}\left(t_{0}\right)\right)\right)\text{,}$$

where *t*_*0*_ – is the time moment of CT saturation occurrence, *i*_*2*_*(t*_*0*_*)* and *i*_*2e*_*(t*_*0*_*)* – are the measured and predicted current values obtained at the *t*_*0*_, *λ(t*_*0*_*)* – is the initial magnetic density at the moment of *t*_*0*_.

Then using (2), (3), (4), which is method A1, the saturated section of measured current can be restored.

The advantage of A3 is its reduced sensitivity to remanent flux density *λ(t*_*0*_*)* in the CT core. One of its disadvantages is its complete dependence on the predicted current values at the beginning of the distorted section. Its other disadvantage is the use of CT parameters.

A main feature of the methods A1–A3 is the need to detect the moment of occurrence of CT saturation. In real conditions, when measurements are received in packages and when they are subject to noise and harmonic components, to determine the moment of saturation occurrence is impossible. With an inaccurate detection of the saturation moment the estimation of the fault current parameters in A2 and A3 is shifted towards bad measurements. As a result, the efficiency of methods A2 and A3 is sharply reduced. Getting bad measurements into A1 negatively affects the calculation of flux density. As a result, there is a sharp deterioration in the operation of the method due to the shape of the magnetization curve (poor conditioning). Table 1 shows comparative analysis of the methods A1-A3.

**Table 1:** Comparative analyses of methods A1-A3.

Reference	Method	Essence	Advantages/Disadvantages
[[10], [11], [12], [13]]	A1	based on the use of CT mathematical model	(+) Robustness to noise and harmonics (−) Highly sensitive to remanent flux density
[[14], [15], [16], [17]]	A2	based on the use of fitting and extrapolating functions	(+) Robustness to remanent flux density (−) Highly sensitive to noise and harmonic components as well as to the moment of saturation occurrence
[[18], [19], [20], [21], [22]]	A3	Combination of previous A1 and A2 methods	(+) Decreased sensitive to remanent flux density (−) Highly sensitive to noise and harmonic components as well as to the moment of saturation occurrence

It results from Table 1 that the more effective method is A3 which contains methods A1 and A2. However, using this method requires the detection of the moment of CT saturation occurrence with high accuracy. The result of computational experiments shows that the small distortion of the moment of the CT saturation can sharply decrease the accuracy of method A3 [7]. The results of another computational experiments show that to determine the moment of CT saturation with high accuracy is impossible [41]. A more detailed description of methods A1-A3 is given in Ref. [7].

The above-described features of the deterministic methods A1-A3 lead to a search of new approaches without reference to both the moment of the CT saturation and remanent flux density. One of them is the use of ANN. The following is an overview and comparative analysis of the existing and proposed ANN-based methods for restoring CT secondary current.

### 2.2 Review of CT compensation methods based on the use of artificial neural networks

The process of restoring the CT current in saturation mode using the ANN will consist of data generation and dataset formation, data preprocessing and training of the ANN, as well as data postprocessing in order to restore the original scale. To solve the CT core saturation problem, as a rule, an ANN is used that requires training with a teacher – each of the input data has its own label at the output. In the context of CT saturation, measured current values are used as input data, and reference current values are used as a teacher.

In [23], a method based on combination of ANN and a fuzzy model is proposed. The network contains 5 layers: input, 3 hidden and output. A distinctive feature of this work from others is the additional inputs on the fourth hidden layer. The mean squared error (MSE) was chosen as the criterion for the quality of the network. There is no information about the number of neurons in the layers in the work.

In [24], a fully connected forwarded ANN is proposed. The number of nodes of the input layer is 20 when sampling rate of the measured current is 20 samples/cycle. The network contains one hidden layer with two neurons. The output layer includes one neuron. There is no description of the work regarding the activation function, the speed of network learning and the criteria for the quality of network operation. The current is restored by sliding the window in one-dimensional increments. This network is designated ANN1.

The advantage of ANN1 is the small number of neurons in the hidden layer and the output layer. However, this leads to a decrease in its flexibility with respect to harmonics in the measured current and the shape of the distorted current.

In [25,26], to restore the current a forwarded ANN architecture is proposed. Next, this architecture will be designated as ANN2. The network contains an input layer with 30 nodes and one neuron on the output layer. It uses two hidden layers with 12 and 8 neurons, respectively. The sigmoidal function was used as the activation function. The sampling rate of the signal was 20 points/period. This means that ANN input layer accepts a current of 1.5 cycles. The paper does not describe the assessment of the quality of the ANN performance.

The positive sides of ANN2 include the size of the input layer (1.5 periods). This helps to quickly train the network and reliably restore distorted current sections. On the other hand, a period and a half of data accumulation leads to a delay in the operation of the RP&EA.

To restore the CT secondary current in Ref. [27] a recurrent ANN with two hidden layers, each of which contains 8 neurons, is proposed. The output of the network contains one neuron. The input layer covers the full current period with two additional nodes. Additional nodes are designed to receive the calculated current values from the network output for *i*_*ANN*_*(n-1)*, *i*_*ANN*_*(n-2)*. All neurons in the network use a sigmoidal activation function.

The positive sides of the network proposed in Ref. [27] include a small number of neurons. The main disadvantage of the work is the small number of weight connections of the output layer. This reduces the flexibility of the model to fault modes with harmonics.

In [28] a recurrent ANN is proposed to solve the problem of current recovery. The network contains two nodes on the input layer, one hidden layer with 6 neurons and one neuron on the output layer. The neurons of the hidden layer use a sigmoidal function multiplied by a scaling factor. The output layer neuron uses a linear activation function. The network on the input takes the values of flux density *λ(n)* and magnetizing current *i*_*μ*_*(n-1)* of CT. The output of the network is a magnetizing current *i*_*μ*_*(n)*.

The absolute advantage of the network suggested in Ref. [28] is the use of a scaling factor in the sigmoidal function and a small number of neurons. The scaling factor increases the flexibility of the neuron activation function. Its main disadvantage is the poor choice of values at the input and output of the network. Both flux density and magnetizing current are calculated quantity the values of which are difficult to obtain in real conditions. These values depend on many factors as well.

To compensate the CT error in the saturation mode in Refs. [29,30], a fully connected forwarded ANN is proposed. The network consists of an input layer with 32 nodes (the full cycle of the current), two hidden layers, respectively, with 10 and 6 neurons and an output layer with one neuron. Both the hidden and the output layers contain a sigmoidal activation function. Next, the method will be denoted ANN3.

The advantages of ANN3 include the optimal number of layers and the number of neurons contained in them, as well as point-by-point current recovery which helps to smooth the calculated curve. Its main disadvantage is the computational load due to the single output neuron. Thus, the restoration of each of the current values causes the need to re-call the network and activate the neurons embedded in it.

In [31], solutions to the problem of current restoration using the ANN are proposed. This method will be denoted as ANN4. The network architecture is fully connected with forward propagation. The network contains an input, two hidden and an output layer. The Rectified Linear Unit (ReLU) was used as an activation function in the work. The number of neurons in the first and second hidden layers was 95 and 90, respectively. The number of input neurons was equal to the sampling rate of the signal of 100 points/cycle.

To increase the effectiveness of network training random selection of initial weight, splitting training data into mini-batches and adaptive Momentum algorithms are used in the work. Also, due to the large number of neurons and nodes of the input layer, intermediate training (autoincoder & autodecoder) was used. The essence of such training is to select more appropriate initial weight.

The positive aspects of the algorithm include its flexibility due to the large number of neurons in the hidden layers and the input layer. This, in addition to saturation of the CT with varying degrees of depth, allows to take into account many other transient modes caused, for example, by overvoltage of the power transformer and saturation of its core. The main disadvantage of ANN4 is the unsuccessful application of the activation function (ReLU) in the neurons of the hidden layer:

(9) $$\left(x\right)=\left\{ \begin{array}{c} 0,x<0\\ x,x\geq 0 \end{array}\right.\text{.}$$

It can be seen from the expression (9) that with negative input values, the neurons output zero, i.e. the negative half-cycle of the measured current is not restored. Another disadvantage of ANN4 is the computational load of the network due to the number of neurons.

In [32], the use of ANN is proposed to reduce the CT error. The architecture of the network is fully interconnected and forwarded ANN. It contains an input, two hidden and an output layer. The input layer contains 27 nodes. The first 20 nodes correspond to one cycle of measured current, the 21st node takes the RMS value of measured current. The remaining 6 nodes take the scaling coefficient of the main, second and fifth harmonics and their phase. The number of neurons of the first and second hidden layers is 12 and 8, respectively. At the output, the network issues the phase and the scaling coefficient of CT. The criterion for the quality of the ANN operation in Ref. [32] is the MSE.

The positive sides of this network include a small number of neurons in the hidden and output layers and nodes of the input layer. However, the network is designed to improve the accuracy of the CT in its normal operating modes.

In [33] to solve the problem of current recovery the use of ANN with a fully connected and forwarded architecture is proposed. The number of nodes of the input layer in this network is equal to the sampling rate of the signal which is 64 points/cycle. Regarding the hidden layers and the output layer, there is no mention in the paper. Instead, a description of a pre-trained network (autoencoder) for noise smoothing is given. The number of hidden layers is 3. The first layer contains 60 neurons, the second and third – 57 and 55, respectively. The network works by sliding the data window in one-dimensional increments. As an activation function in neurons, a sigmoidal function with centered characteristic relative to coordinate system is used. The criterion for the quality of network operation is the MSE.

The positive aspects of the proposed network in Ref. [33] include the smoothing the noise components. However, the number of neurons in the first part of the network – 60, 57 and 55 – strongly affects the speed of the algorithm.

The main positive side of all the considered networks is the absence of the need to solve CT saturation detection and distinguish unsaturated section for saturated one.

The main disadvantage common to all the considered networks is data scaling based on the use of the maximum sample value. With this scaling, it becomes necessary to search for the maximum current value across the entire interval of generated data. If it is located at the end of the interval, there is a delay in restoring the current. Otherwise, the range of scaled data exceeds ±1, which negatively affects the result of the model. Another disadvantage of most of the considered networks is the first cycle of current data after fault, because based on them, ANN1 – ANN4 give a predicted value of the current for the next period.

The properties of the above-described networks are shown in Table 2.

**Table 2:** Comparative table of current recovery methods with different architectures and sizes of ANN.

Literature	Network architecture	Hidden layers	Neurons of hidden layers	Activation function	The output neuron	Advantages and disadvantages
[23]	FANN2	4	–	–	1	(+) Flexibility due to the number of hidden layers (−) Computing load
[24]	FANN	1	*h*_*1*_ *=* *2*	–	1	(+) A small number of neurons (−) Lack of flexibility
[25,26]	FANN	2	*h*_*1*_ *=* *12,* *h*_*2*_ *=* *8*	Sigmoid	1	(+) Large current coverage at the network input (−) Relatively long delay of the protection system
[27]	RNN	2	*h*_*1*_ *=* *8,* *h*_*2*_ *=* *8*	Sigmoid	1	(+) The optimal number of neurons in the hidden layers (−) A single neuron at the network output
[28]	RNN	1	*h*_*1*_ *=* *6*	Sigmoid	1	(+) Additional increase in network flexibility due to the scaling factor in activation functions (−) The input/output values used (density and magnetizing current)
[29,30]	FANN	2	*h*_*1*_ *=* *10,* *h*_*2*_ *=* *6*	Sigmoid	1	(+) The optimal number of hidden layers and neurons in them (−) Computational load due to a single output neuron
[31]	FANN	2	*h*_*1*_ *=* *95,* *h*_*2*_ *=* *90*	ReLU	–	(+) Flexibility due to the large number of neurons (−) High computational load
[32]	FANN	2	*h*_*1*_ *=* *12,* *h*_*2*_ *=* *8*	Sigmoid	1	(+) Incoming network nodes contain instantaneous and RMS current values (−) The network is designed to compensate for the CT error in normal operation modes
[33]	FANN	–	–	Sigmoid	1	(+) Noise reduction and selection of the most appropriate initial weights due to autoencoder (−) High computational load due to the presence of an autoencoder

∗FANN is a fully interconnected neural network with forward propagation, FANN2 is an FANN with two inputs in the 4th layer, RNN is a recurrent neural network.

## 3 Method

### 3.1 Suggested ANN

To restore the distorted shape of the current curve obtained due to saturation of the CT core, a forwarded ANN with a fully connected architecture was selected. It includes an input, two hidden and an output layer. The input and output layers of the network receive and output a 1 × 32 line vector (measured values of the current with CT saturation and its restored values, respectively). The reconstructed values should have a significantly lower error compared to the measured values, since the latter were distorted due to CT core saturation. The number of neurons of each of the hidden layers *h*_*1*_ and *h*_*2*_ will vary from 5 to 15. As a result, a set of networks is obtained, and the most effective network is chosen to restore the CT current. The search for the most suitable network model will be given at the end of this section.

One of the main factors determining the quality of the ANN and its flexibility in training and subsequent use is the activation function. It is used in neurons of all layers. The choice of this function depends on the type of task being solved. In the context of the task of restoring instantaneous current values during CT saturation, it should be considered that the current curve varies in the positive and negative regions. This means that the area of change in the activation function should be in the I and III quadrants. Based on the mentioned statement, when solving the problem of restoring current in all neurons of all layers of the ANN a modified sigmoidal function was laid down:

(10) $$f\left(x\right)=\frac{2}{1+\exp\left(-x\right)}-1\text{.}$$

**Fig. 3:**
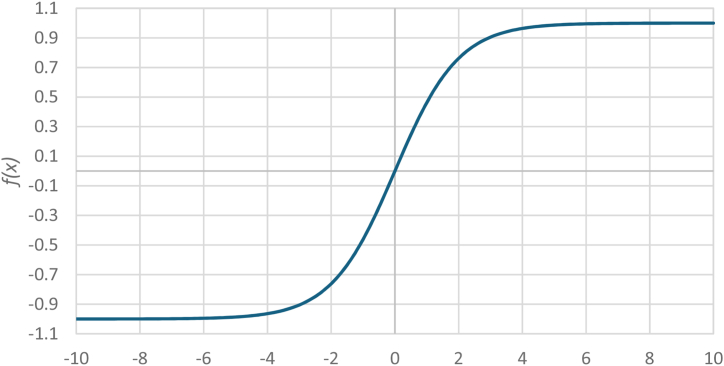
Graph of the sigmoidal function after centering.

A modification of this function is to center its graph relative to the origin. As a result, the scope of the specified function expands and covers both the first and third quadrants. Thus, it is possible to restore the positive (first quadrant) and negative (third quadrant) half-cycles of the measured current. Fig. 3 shows a graph of the sigmoidal function after its centering.

Fig. 3 also shows that the extremum of the activation function is limited to the range [−1; 1]. This means that the input values of this function must be such that its output value is in a linear section. Otherwise, in the process of learning the ANN, the neurons are quickly “saturated” and the learning ability of the network decreases sharply. This generally requires standardization and normalization of data.

### 3.2 Data preprocessing

Standardization is designed to convert a data distribution to a zero-mean distribution. When the distorted current is restored at the real-time working, the task of standardization disappears. This is due to the fact that when the ANN is working with a sliding data window, the average value of the data at both the input and output of the ANN changes. Next point is data normalization. The main purpose of normalization is to bring data with different values and different ranges of values to a single scale. In particular, to solve the problem of restoring distorted current, it is necessary to bring the current data obtained under various fault modes to a single scale, as a result the values of each training sample will be in the range [−1; 1]. Thus, when normalizing data the following range of tasks is solved for the ANN: the influence of the numerical spread of current data is eliminated; the values of the normalized data fall into the linear domain of the sigmoidal function used in each neuron, which increases the efficiency of the ANN at the learning stage; the gradient value decreases during the training of the ANN, which avoids skipping the global extremum of the loss function.

There are many methods for normalizing data to a single range at the data preprocessing stage. The most used ones are Z-normalization, Min-Max- and Max-normalization. The effectiveness of these methods depends on the type of problem being solved and the nature of the data.

It is worth noting that when using these normalization methods, the task of restoring the CT current becomes more complicated. The effectiveness of ANN training is deteriorating, and its accuracy is decreasing. This is due to the fact that the ANN must work at the real-time working process by a sliding data window. When the data window is shifted, the main indicators of training samples (minimum, maximum and average values) change. Thus, when a new data packet arrives at the ANN input, the old “not discarded” packets repeatedly change the scale, which negatively affects the result of the ANN operation.

**Fig. 4:**
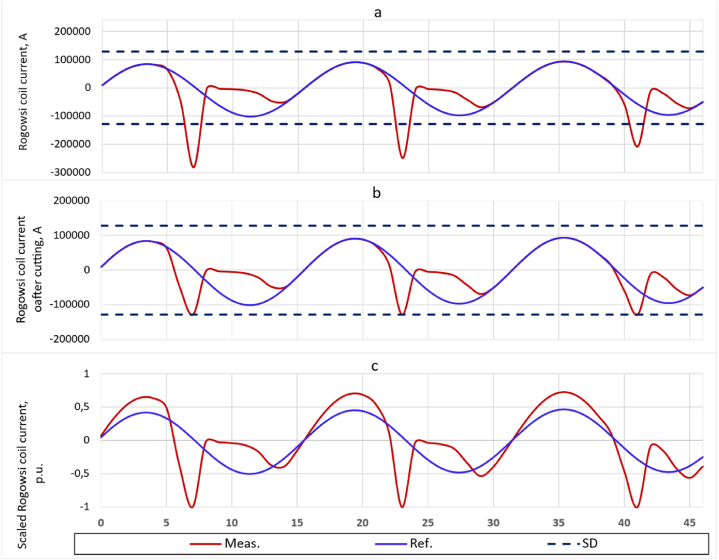
Current curves at the output of the Rogovsky coil and standard deviation the measured current: (a) and (b) – before and after the cut, respectively, (c) – after scaling.

Since the measured current values are obtained at the output of the Rogovsky coil, sharp emissions occur when the CT is saturated, Fig. 4. In this case, it is possible to obtain the standard deviation (SD) σ of the first period, taking into account the saturation of the CT obtained since the occurrence of the fault. And then, based on σ, scale all data, regardless of their indexing by package and cycle. To illustrate what has been said, Fig. 4, *a* shows the current curves and σ values for the first period of the measured current obtained at the output of the Rogovsky coil at CT saturation. The measured and reference currents are highlighted in color. The black dotted lines show the boundaries *σ* of the measured current values. The clipping of sudden bursts of measured current caused by CT saturation is performed as:

(11) $$i\left(t\right)=\left\{ \begin{array}{c} i\left(t\right),i\left(t\right)<\kappa ∙\sigma \\ \kappa ∙\sigma ,i\left(t\right)\geq \kappa ∙\sigma \end{array}\right.\text{,}$$

where *σ* – standard deviation of the first cycle of the measured current; *κ* – reliability factor, *κ* = 1.5.

After applying formula (11), the emissions of the measured current are cut off, as shown in Fig. 4, *b*.

Further, the data obtained as a result of the slice must be divided by the value of the standard deviation and the reliability coefficient κ.

(12) $$i_{scaled}\left(t\right)=\frac{i\left(t\right)}{\kappa ∙\sigma }\text{,}$$

where *i*_*scaled*_*(t)* – scaled measured current data.

Thus, the input data of the ANN goes through the normalization procedure.

Now there is a need to normalize the reference (required) data. Since the reference data is not subject to distortion caused by CT saturation, it is enough to divide each of them by its absolute maximum value obtained from the first period. After that, reference data with an absolute maximum value of 1 is obtained. To increase the effectiveness of ANN training, it is advisable to divide the normalized values of the reference current by 2. In this case, in the process of learning the ANN, the search for the extremum of the objective function is performed in the linear domain of the activation function, which increases the flexibility and speed of learning the ANN. The input and reference data of the ANN are shown in Fig. 4, *c*. It can be seen from the figure that the values of the extremes of the reference curve do not exceed ±0.5.

This is how data is preprocessed and prepared for ANN training.

After training the network at its output, it is possible to obtain restored current values in the range ±0.5. Therefore, there is a need for reverse scaling of the data. For this purpose, calculated and measured current values are used, obtained within 2 ms from the moment of occurrence of a fault.

(13) $$K=\frac{\sum_{k=1}^{N}i_{meas}\left(k\right)}{\sum_{k=1}^{N}i_{ANN}\left(k\right)}\text{,}$$

where *i*_*meas*_*(k)* and *i*_*ANN*_*(k)* are measured and calculated values of the *k*-th reference current; *N* is the number of points obtained during 2 ms from the moment of occurrence of a fault.

Knowing the data scaling factor *K*, it is possible to restore the original scale of the measured current curves.

### 3.3 Data used

When using ANN in the context of CT error compensation under core saturation conditions, the variation of factors that affect the depth and nature of CT saturation is required. Such factors are: the multiplicity of the fault, the moment of fault occurrence, the DC component of the fault, the secondary CT load and the remanent flux density of the core. These factors, excluding secondary load, are completely random.

To take into account the parameters connected to the fault, a series of current curves consisting of two modes:

– normal

(14) $$i_{1}\left(t\right)=I_{m1}\;\sin\left(\omega t\right)$$

– and fault:

(15) $$i_{2}\left(t\right)=kI_{m1}\;\sin\left(\omega t+\varphi \right)+I_{m2}e^{-\frac{t-t_{0}}{T}}$$

were submitted for the CT input.

In (15) *k* and *I_m1_* are the multiplicity and the amplitude of AC component of the current, respectively; *φ* is the angle of fault occurrence; *I_m__2_* and *T* are the initial value and decay rate of the DC component of the current in fault conditions, respectively; *t*_*0*_ is the moment of the mode transition from one characteristic to another; *ω* is the cyclic frequency.

The initial value of the DC component of the current is related to the angle of fault occurrence *φ*, since it is obtained from the condition of current constancy at the time of changing the mode *t*_*0*_.

(16) $$I_{m1}\;\sin\;\omega t_{0}=kI_{m1}\;\sin\left(\omega t_{0}+\varphi \right)+I_{m2}\text{.}$$

Thus, the initial value of the DC component:

(17) $$I_{m2}=I_{m1}\;\sin\;\omega t_{0}\;–\;kI_{m1}\;\sin\left(\omega t_{0}+\varphi \right)\text{.}$$

To take into account the fault parameters affecting CT core saturation the following was performed:

• the fault multiplicity varied in the range *k* = 10 ÷ 55 in increments of 5;
• the angle of fault occurrence varied in the range *φ* = −90° ÷ 110° in increments of 5°.

As a result, 400 fault modes were simulated.

Next factors are the remanent flux density*λ(t_0_)* and the secondary load. The effect of *λ(t_0_)* on CT saturation is identical to the effect of network parameters – the time of saturation occurrence. The secondary load, in addition to the time of saturation occurrence, affects the shape of the distorted current curve as well. This is important when solving a problem by applying ANN methods. To take into account these last two factors (remanent density and secondary load), previously synthesized current curves (400 fault modes) were supplied to the CT input with consideration of following:

• the remanent density varied in the range of *λ* = 0 ÷ 1.8 *T* in increment of 0.2 *T*;
• the active secondary load varied in the range of *R*_*n*_ = 0.96 ÷ 1.6 *Ω* in increment of 0.2 *Ω*;
• the reactive secondary load varied in the range of *X*_*n*_ = 0 ÷ 0.8 *Ω* in increment of 0.2 *Ω.*

Thus, taking into account all the variable parameters, the total number of simulated modes was:

(18) $$N=N_{1}∙N_{2}∙N_{3}∙\left(N_{4}+N_{5}\right)=10∙40∙10∙\left(6+5\right)=44,000\text{.}$$

In (18) *N*_*1*_ and *N*_*2*_ are the total number of fault multiplicity and fault occurrence angle, respectively, *N*_*3*_ is the number of remanent flux densities, *N*_*4*_ and *N*_*5*_ are the total number of active and reactive secondary loads, respectively.

**Fig. 5:**
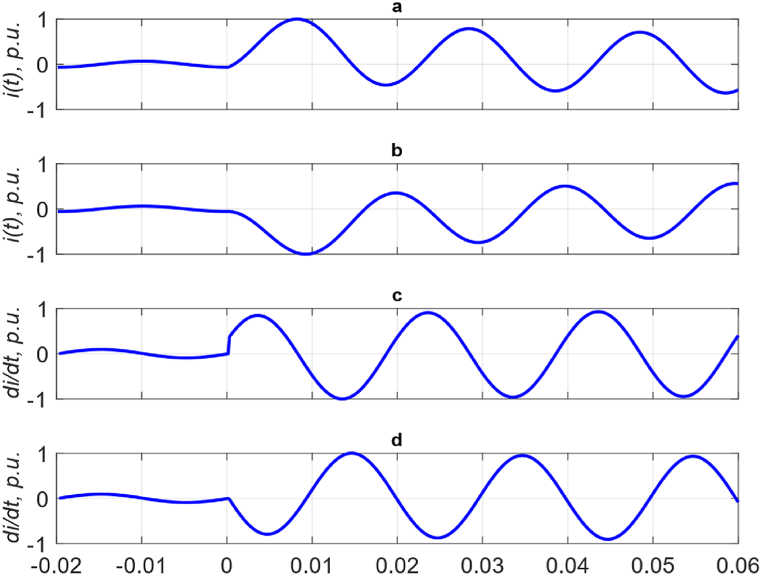
Current curves of normal and faults modes: *a* and *b* are fault with angle −40° and 80°, respectively; *c* and *d* are the first derivative of graphs *a* and *b*, respectively.

The current curves at the output of the CT and the corresponding one at the output of the Rogovsky coil are shown in Fig. 5. The current curves consist of normal (up to the moment *t*_*0*_ = 0) and emergency (after the moment *t*_*0*_ = 0) modes. Graphs *a* and *b* show CT currents at the fault occurrence with angles *φ* = −40° and *φ* = 80°, respectively. It can be seen from graphs *a* and *b* in Fig. 5 that, depending on the value of *φ*, the sign and the proportion of the DC component of the fault current may vary. Graphs *c* and *d* show the current curves at the output of the Rogovsky coil, which correspond to graphs *a* and *b*.

Since the CT measurements are converted into a current derivative in the Rogovsky coil, by numerical differentiation of the first order, the generated currents are converted into a derivative by numerical differentiation of the first order, Fig. 5, graphs *c* and *d*. Thus, the simulation of the operation of the Rogovsky coil is performed.

**Fig. 6:**
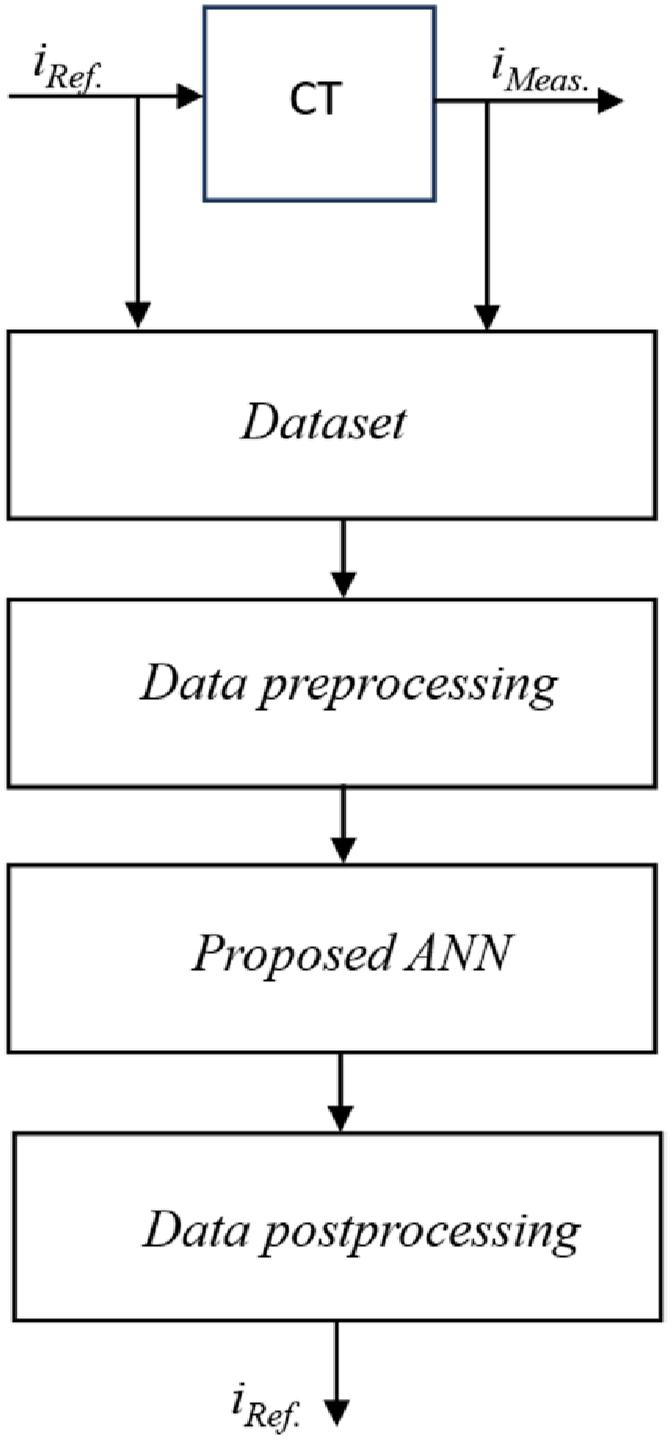
CT current recovery procedure based on the proposed ANN.

The procedure for data recovery using the ANN proposed within the work is graphically presented in Fig. 6.

Fig. 6 shows that the data is generated and goes through the data preprocessing stage using expressions (11) and (12). After that, the distorted sections of the measured current are restored, and the initial scale of the measured current curves is reversed according to the expression (13).

## 4 Results

### 4.1 Choosing a neural network model

**Fig. 7:**
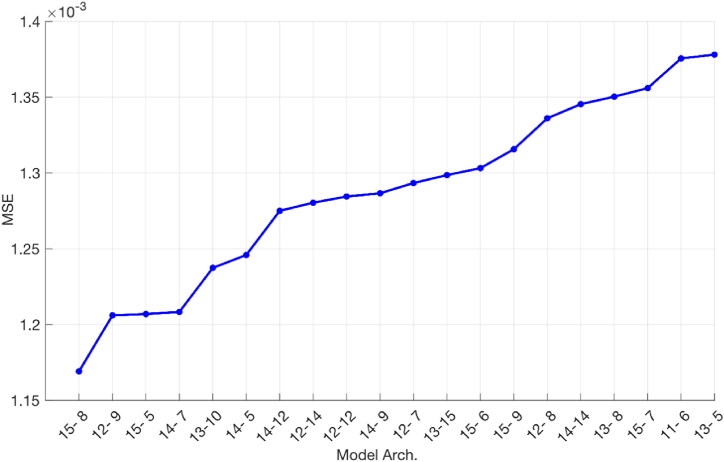
The most suitable ANN models for CT current recovery.

After choosing the appropriate method of pre- and post-processing of data, justification and selection of the activation function and synthesis of CT saturation modes, it is necessary to choose the appropriate ANN model. To do this, a series of calculations is performed with a variation in the number of neurons of hidden layers in the range *h*_*1*_ = *h*_*2*_ = 5 ÷ 15. At the same time, the “width” of the input and output layers was fixed. With the variation of neurons *h*_*1*_ and *h*_*2*_, 121 ANN models were obtained. The number of epochs was Epoch = 500. As a result, the obtained models were sorted by the lost functions values in the form of MSE. The first 20 of them are shown in Fig. 7, where the numbers of neurons *h*_*1*_ and *h*_*2*_ are laid along the *Ox* axis, and the values of MSE are laid along the *Oy* axis.

**Fig. 8:**
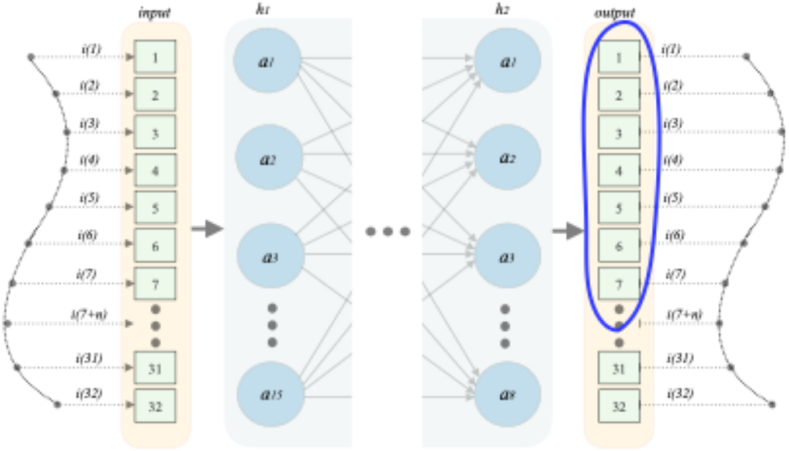
Architecture of the selected neural network.

From the graph in Fig. 7 it can be seen that the most suitable ANN is a neural network with *h*_*1*_ = 15 and *h*_*2*_ = 8 neurons. Accordingly, this ANN is then used as a model for restoring current and denoted as ANN5. Thus, the network model contains 4 layers and is shown in Fig. 8. In this figure it is seen that the sampling rate of the signal is 32 samples/cycle.

It is worth noting that at the stage of training, validation and the first start-up during operation, the network receives and outputs current data from one cycle, Fig. 8. The data sliding step is 5 ms. In other words, there is a batch update of data every 5 ms. The mentioned can analytically be represented as:

(19) $$\left\{{\left\{i_{k}^{meas};i_{k}^{ANN}\right\}}_{k=1}^{32};\;{\left\{i_{k}^{meas};i_{k}^{ANN}\right\}}_{k=m+1}^{32+m};...{\left\{i_{k}^{meas};i_{k}^{ANN}\right\}}_{k=n∙m+1}^{32+n∙m}\;|\;m=8;n=1,2,3,\ldots \;k\right\}\text{,}$$

where *m* and *n* are the number of measurements in one package and the package number, respectively.

At the operational stage, during subsequent launches, the size of the output layer is cut off by removing its neurons, which produce calculated current values of 3 old packets (1 ÷ 15 ms). This is shown in Fig. 8 by outlined in blue. As a result, the number of neurons in the output layer is equal to the data of the last packet received at the rate of the process. Thus, the network dimension is reduced and has a positive effect on the computing load of microprocessor devices. Science input and output data are updated every 5 ms, to restore the current of one cycle the ANN is started 4 times regardless of the sampling rate of the signal.

### 4.2 Approbation and comparative analysis of current recovery methods based on ANN

In this section, the most promising from the point of view of their practical application of the considered methods of current recovery based on the use of ANN1-ANN4 and their comparative analysis with the proposed ANN5 are tested.

The duration of each mode is 3 periods of power frequency current. The properties of the proposed and analyzed ANN-based models are shown in Table 3.

**Table 3:** Properties of the analyzed ANN.

ANN	The size of the input layer	Number of h layers	Neurons of h layers	Activation function
ANN1	1 period	1	*h*_*1*_ = 2	$y=\frac{2}{1+e^{-x}}-1$
ANN2	1.5 periods	2	*h*_*1*_ = 12, *h*_*2*_ = 8	$y=\frac{2}{1+e^{-x}}-1$
ANN3	1 period	2	*h*_*1*_ = 10, *h*_*2*_ = 6	$y=\frac{2}{1+e^{-x}}-1$
ANN4	1 period	2	*h*_*1*_ = 95, *h*_*2*_ = 90	$y=\left\{ \begin{array}{c} 0,x<0\\ x,x\geq 0 \end{array}\right.$
Proposed ANN5	1 period	2	*h*_*1*_ = 15, *h*_*2*_ = 8	$y=\frac{2}{1+e^{-x}}-1$

∗*h* – designation of hidden layers.

More detailed information about the other hyperparameters of the networks being tested is given in Table 4.

**Table 4:** Table of the initial data of the tested ANN-based models.

Name of the parameter	The value of the parameter
Learning rate	η = 0,01
Total data size (number of short circuit modes)	Dataset = 44000
The size of the training data	Dataset_train = 0,7·Dataset
The size of the verification data	Dataset_valid = 0,15·Dataset
The size of the text data	Dataset_test = 0,15·Dataset
Number of epochs	Epoch = 500
Criteria for the quality of the network	mse
Mini-batch size	S = 128
Window sliding step	d = 1

One epoch includes running all pairs of Dataset_train data with sliding the data window into one dimension (32 times for the full period for ANN1-ANN4). Based on the above, the size of the epoch was assumed to be 500.

**Fig. 9:**
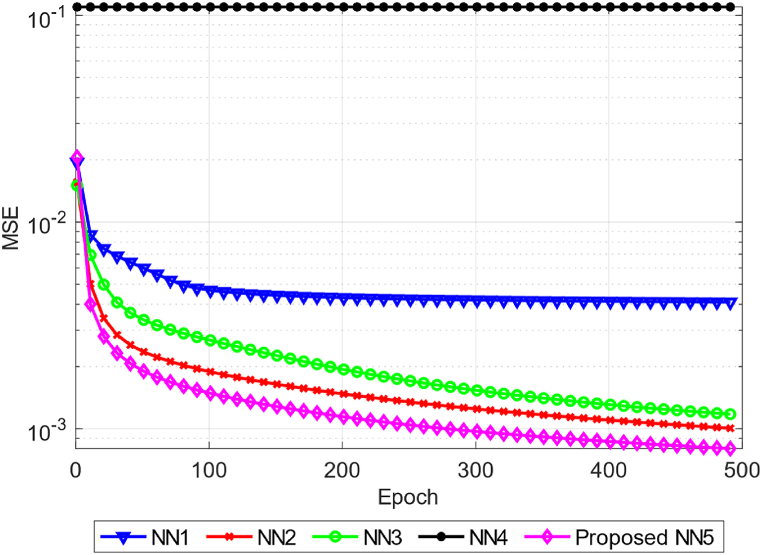
The average error is ANN1-ANN5 for all short circuit test modes over 500 epochs.

Fig. 9 shows the dependence of the ANN1-ANN5 efficiency in the form of MSE on the number of epochs. The ANN1-ANN5 in the figure are labeled as NN1-NN5. The calculation of the average MSE of each of the considered ANN was performed on the basis of all verification data. It can be seen from the figure that ANN4 has not been able to increase the efficiency of its work in 500 epochs. This is caused by an unsuccessful choice of the ReLU activation function. It is not possible to restore the values of the reverse half-cycle current using this function. ANN2 with two hidden layers with neurons *h*_*1*_ = 12 and *h*_*2*_ = 8, respectively, proved to be the most effective. After ANN2, ANN3 and ANN1 are effective. The behavior of ANN1 with one hidden layer containing 2 neurons is interesting. After the hundredth epoch, the efficiency of this network stops growing. This means that either the number of neurons in the hidden layer or the number of layers is not enough to solve the problem of restoring the CT current.

Table 5 shows the MSE values for each of the models over 500 epochs. As the calculation results show, among the analyzed models (ANN1-ANN4), ANN2 with two hidden layers of dimension *h*_*1*_ = 12 and *h*_*2*_ = 8 proved to be the most effective. However, the efficiency of the proposed ANN5 architecture is on average is MSE = 0.0013 while the efficiency of ANN2 on average is MSE = 0.017 meaning that ANN5 efficiency 1.3 times greater than the efficiency of ANN2. Over 500 epochs, ANN5 approximated a series of current curves relative to ANN2 1.25 times more accurately.

**Table 5:** Efficiency of ANN1-ANN5 over 500 epochs.

Model	Average MSE, p.u.	Minimum MSE, p.u.
ANN1	0.0047	0.0041
ANN2	0.0017	0.001
ANN3	0.0022	0.0012
ANN4	0.1097	0.1097
Proposed ANN5	0.0013	0.0008

The effectiveness of the proposed ANN5 is explained by the dimensionality of its input and output layers. The coverage of the full period both at the input and at the input allows to more accurately select the weight connections in the model.

**Fig. 10:**
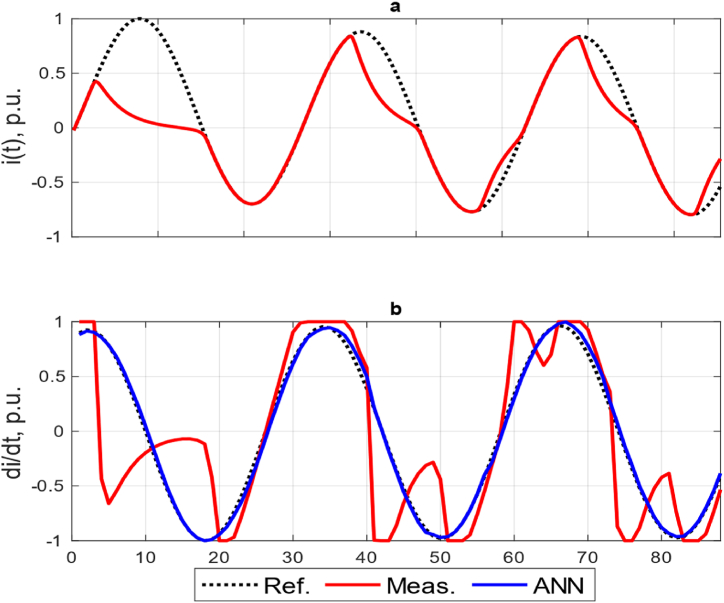
Curves of the reference, measured and calculated currents: (a) At the output of the CT; (b) At the output of the Rogovsky coil and the proposed ANN.

Because due to the architecture of ANN1-ANN4, the original scale of currents will be restored, they will not be considered further. To illustrate the operation of the proposed ANN5, Fig. 10 shows the current curves at CT saturation. Graph *a* shows the reference and measured values of the currents at the output of the CT, graph b shows the corresponding currents at the output of the Rogovsky coil and the proposed ANN.

**Fig. 11:**
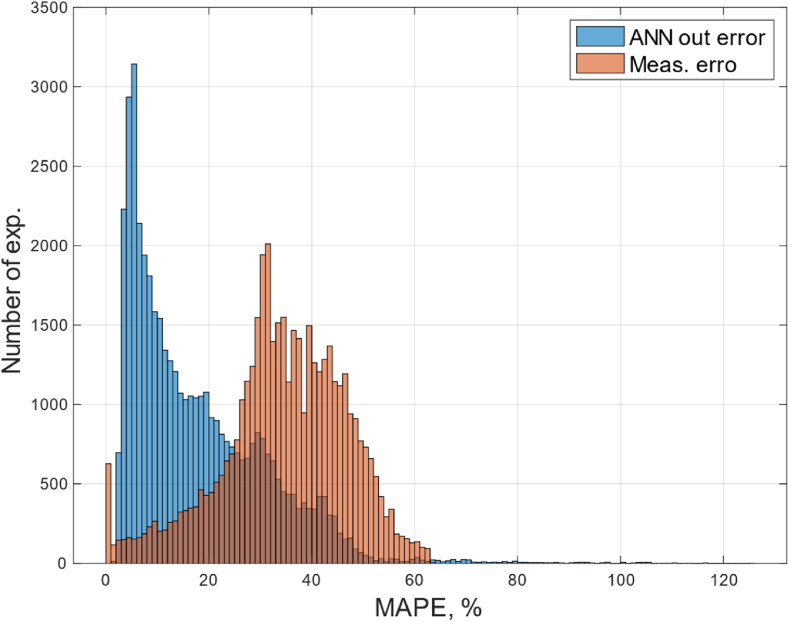
Error distribution diagram before and after the application of the proposed ANN.

Since the proposed ANN5 is an approximator, not a forecasting model, all 44,000 modes can be taken into account at the testing stage. Fig. 11 shows the distribution of errors measured and calculated at the output of the proposed ANN5 currents for all 44,000 modes. The *Ox* axis contains current errors in the form of mean absolute percentage error (MAPE), and the *Oy* axis contains the number of modes. The error spread of more than 100 % is caused by an erroneous restoration of the original data scale, obtained as a result of deep saturation of the CT or current reversal when a short circuit occurs.

## 5 Conclusions

The analyzes of deterministic methods has been proposed in the paper. The advantages and disadvantages of the mentioned methods are shown, and the use of artificial neural network methods is justified. It has been determined that during solving the CT saturation problem by using ANN the segmentation of the measured current into distorted and undistorted sections is not required. A detailed analysis of ANN methods with computational experiment is presented. It has been established that existing ANN-based models use direct scaling and reverse data recovery methods when restoring current, which excludes the possibility of their practical application.

It has been shown that both the number of neurons and the activation function are important components of the ANN during solving CT core saturation problem. For example, it is shown that when replacing the ReLU function in hidden layers with a sigmoidal function, the efficiency of the ANN increases dramatically. It is established that when using neural networks, it is necessary to use a sigmoidal activation function with a shift of its graph to the center of the coordinate system.

A method adapted to real conditions for scaling and reverse scaling of data at the stage of pre- and post-processing of data is proposed to solve the problem of restoring the CT current. The results of computational experiments for current recovery methods based on the use of ANN are presented. It is shown that the ANN with coverage of the full period of the input and output current approximates the shape of the current curve on average 1.3 times more accurately. It was found that an ANN with two hidden layers at *h*_*1*_ > *h*_*2*_ is the most effective than an ANN with *h*_*1*_ < *h*_*2*_.

Have read and agreed to the published version of the manuscript.

## Funding

No sources.

## Data availability statement

Data will be made available on request.

## CRediT authorship contribution statement

**Ismoil Odinaev:** Writing – original draft, Visualization, Validation, Software, Methodology, Investigation, Formal analysis, Data curation, Conceptualization. **Abdel-Haleem Abdel-Aty:** Writing – original draft, Supervision, Project administration, Funding acquisition, Formal analysis, Data curation, Conceptualization. **Andrey Pazderin:** Writing – review & editing, Validation, Resources, Project administration, Investigation, Funding acquisition, Formal analysis, Data curation. **Murodbek Safaraliev:** Writing – review & editing, Writing – original draft, Supervision, Software, Resources, Methodology, Investigation, Conceptualization. **Pavel Matrenin:** Writing – review & editing, Software, Methodology, Investigation, Conceptualization. **Mihail Senyuk:** Writing – original draft, Validation, Software, Methodology, Formal analysis, Data curation, Conceptualization. **Amir Abdel Menaem:** Writing – review & editing, Validation, Resources, Methodology, Formal analysis, Data curation, Conceptualization. **Mohammad Kanan:** Writing – original draft, Visualization, Resources, Project administration, Methodology, Investigation, Funding acquisition, Data curation, Conceptualization.

## Declaration of competing interest

The authors declare that they have no known competing financial interests or personal relationships that could have appeared to influence the work reported in this paper.

## References

[1] Senyuk M., Safaraliev M., Pazderin A., Pichugova O., Zicmane I., Beryozkina S. (2023). Methodology for power systems' emergency control based on deep learning and synchronized measurements. Mathematics.

[2] Senyuk M., Beryozkina S., Safaraliev M., Pazderin A., Odinaev I., Klassen V., Savosina A., Kamalov F. (2024). Bulk power systems emergency control based on machine learning algorithms and phasor measurement units data: a state-of-the-art review. Energies.

[3] Senyuk M., Safaraliev M., Kamalov F., Sulieman H. (2023). Power system transient stability assessment based on machine learning algorithms and grid topology. Mathematics.

[4] Senyuk M., Safaraliev M., Gulakhmadov A., Ahyoev J. (2022). Application of the conditional optimization method for the synthesis of the law of emergency control of a synchronous generator steam turbine operating in a complex-closed configuration power system. Mathematics.

[5] Zegler G. (2005).

[6] das Guerra Fernandes Guerra F., Santos Mota W. (2007). Current transformer model. IEEE Trans. Power Deliv..

[7] Odinaev I., Gulakhmadov A., Murzin P., Tavlintsev A., Semenenko S., Kokorin E., Safaraliev M., Chen X. (2021). Comparison of mathematical methods for compensating a current signal under current transformers saturation conditions. Sensors.

[8] Stano E. (2021). The method to determine the turns ratio correction of the inductive current transformer. Energies.

[9] EC61869-2-2012 (2012). Part 2: Additional Requirements for Current Transformers.

[10] Kang Y., Kang S., Park J., Johns A., Aggarwal R. (1996). Development and hardware implementation of a compensating algorithm for the secondary current of current transformers. IEEE Proc. Electr. Power Appl. Inst. Eng. Technol. (IET).

[11] Kang Y., Park J., Kang S., Johns A., Aggarwal R. (1997). An algorithm for compensating secondary currents of current transformers. IEEE Trans. Power Deliv..

[12] Locci N., Muscas C. (2000). A digital compensation method for improving current transformer accuracy. IEEE Trans. Power Deliv..

[13] Locci N., Muscas C. (2001). Hysteresis and eddy currents compensation in current transformers. IEEE Trans. Power Deliv..

[14] Pan J., Vu K., Hu Y. (2004). An efficient compensation algorithm for current transformer saturation effects. IEEE Trans. Power Deliv..

[15] Macieira G., Coelho A. (2017). Evaluation of numerical time overcurrent relay performance for current transformer saturationcompensation methods. Elec. Power Syst. Res..

[16] Haghjoo F., Pak M.H. (2016). Compensation of CT distorted secondary current waveform in online conditions. IEEE Trans. Power Deliv..

[17] Wiszniewski A., Rebizant W., Schiel L. (2008). Correction of current transformer transient performance. IEEE Trans. Power Deliv..

[18] Shi D.Y., Buse J., Wu Q.H., Jiang L. (2010). Proceedings of the 2010 IEEEPES Innovative Smart Grid Technologies Conference Europe.

[19] Shi D., Buse J., Wu Q., Guo C. (2013). Current transformer saturation compensation based on a partial nonlinear model. Electr. PowerSyst. Res..

[20] Kang Y.C., Lim U.J., Kang S.H., Crossley P. (2004). Compensation of the distortion in the secondary current caused by saturation and remanence in a CT. IEEE Trans. Power Deliv..

[21] Kang Y., Lim U., Kang S. (2005). Compensating algorithm suitable for use with measurement-type current transformers for protection. IEE Proc. Generat. Transm. Distrib..

[22] Hajipour E., Vakilian M., Sanaye-Pasand M. (2015). Current-transformer saturation compensation for transformer differential relays. IEEE Trans. Power Deliv..

[23] Erenturk K. (2009). ANFIS-based compensation algorithm for current-transformer saturation effects. IEEE Trans. Power Deliv..

[24] Cummins J., Yu D., Kojovic L. (2000). Simplified artificial neural network structure with the current transformer saturation detector provides a good estimate of primary currents. Proceedings of the 2000 Power Engineering Society Summer Meeting (Cat.No.00CH37134).

[25] Khorashadi-Zadeh, H.; Sanaye-Pasand, M. An ANN based algorithm for correction of saturated CT secondary current. InProceedings of the 39th International Universities Power Engineering Conference, 2004. UPEC 2004, Bristol, UK, 6–8 September2004; Volume vol. 1, pp. 468–472.

[26] Khorashadi-Zadeh H., Sanaye-Pasand M. (2006). Correction of saturated current transformers secondary current using ANNs. IEEE Trans. Power Deliv..

[27] Saha M., Izykowski J., Lukowicz M., Rosolowskiz E. (9–12 April 2001). Proceedings of the 2001 Seventh International Conference on Developments in Power SystemProtection (IEE).

[28] Baoming G., de Almeida A., Ferreira Fernando J.T. (2006). Estimation of primary current in saturated current transformer using flexible neural network. Trans. Inst. Meas. Control.

[29] Yu D., Cummins J., Wang Z., Yoon H.J., Kojovic L., Stone D. (1999). Neural network for current transformer saturation correction. Proceedings of the 1999 IEEE Transmission and Distribution Conference (Cat. No. 99CH36333).

[30] Yu D., Cummins J., Wang Z., Yoon H.J., Kojovic L. (2001). Correction of current transformer distorted secondary currents due to saturation using artificial neural networks. IEEE Trans. Power Deliv..

[31] Key S., Vattanak S., Sun-Woo L., Chang-Sung K., Soon-Ryul N. (2019).

[32] Ballal M., Wath M., Suryawanshi H. (2019). A novel approach for the error correction of CT in the presence of harmonic distortion. IEEE Trans. Instrum. Meas..

[33] Key S., Kang S., Lee N., Nam S. (2021). Bayesian deep neural network to compensate for current transformer saturation. IEEE Access.

[34] Dashti H., Sanaye-Pasand M., Davarpanah M. (2007). Proceedings of the 2007 42nd International Universities Power Engineering Conference.

[35] Lin G., Song Q., Zhang D., Pan F., Wang L. (2017). Proceedings of the 2017 4th International Conference on Systems and Informatics (ICSAI).

[36] Herlender J., Izykowski J., Solak K. (2020). Compensation of the current transformer saturation effects for transmission line fault location with impedance-differential relay. Elec. Power Syst. Res..

[37] Yang L., Zhao J., Crossley P., Li K. (2010). Proceedings of the 2010 International Conference on Electrical and Control Engineering.

[38] Bahari S., Hasani T., Sevedi H. (31 December–1 January 2019). Proceedings of the 2020 14th International Conference on Protection and Automation of PowerSystems (IPAPS).

[39] Behi D., Allahbakhshi M., Bagheri A., Tajdinian M. (2–4 May 2017). Proceedings of the 2017 Iranian Conference on Electrical Engineering.

[40] Hong C., Haifeng L., Hui J., Jianchun P., Chun H. (2017). Proceedings of the 2017 9th International Conference on Measuring Technology and Mechatronics Automation (ICMTMA).

[41] Odinaev I., Pazderin A.V., Murzin P.V., Tashchilin V.A., Samoylenko V.O., Ghoziev B. (2021). Detection of the initial region of the current transformer core saturation. Renewable Energy and Power Quality Journal.

[42] Rumiantsev Y. (2023).

[43] Odinaev I., Pazderin A., Safaraliev M., Kamalov F., Senyuk M., Gubin P. (2024). Detection of current transformer saturation based on machine learning. Mathematics.

